# Sensor Fault Diagnosis Method Based on Rotor Slip Applied to Induction Motor Drive

**DOI:** 10.3390/s22228636

**Published:** 2022-11-09

**Authors:** Cuong Dinh Tran, Martin Kuchar, Martin Sobek, Vojtech Sotola, Bach Hoang Dinh

**Affiliations:** 1 Power System Optimization Research Group, Faculty of Electrical and Electronics Engineering, Ton Duc Thang University, Ho Chi Minh City 700000, Vietnam; 2 Department of Electronics, Faculty of Electrical Engineering and Computer Science, VSB-Technical University of Ostrava, 708 00 Ostrava, Czech Republic

**Keywords:** current sensorless, fault diagnosis, fault-tolerant control, FOC, speed sensorless

## Abstract

A novel diagnosis method based on the rotor slip is proposed in the paper to correctly detect current and speed sensor failures during the induction motor drive (IMD) operation. In order to enhance reliability and avoid confusion in the diagnosis algorithm due to the influence of measured signal quality, each fault type is determined in a priority order defined by the diagnosis method. Based on the features of the IMD applying the field-oriented control (FOC) technique, an innovative model uses the measured currents and reference speed as the input signals to estimate the rotor slip for the current sensor fault detection. After verifying the quality of the feedback of the current signals, a speed sensor fault function is continued, and performs according to relations among the reference speed, estimated speed based on the sliding mode method, and measured rotor speeds. Finally, the estimated quantities are selected to replace the wrong measured current or speed signals. The feasibility of the proposed approach is verified by simulations using Matlab-Simulink software as well as by practical experiments using an IMD prototype with a rated power of 2.2 kW and a DSC-TMS320F28335-based control system. The obtained simulation and experimental results demonstrated the feasibility, effectiveness, and reliability of the proposed diagnosis technique in detecting sensor failures and maintaining the stable operation of the IMD.

## 1. Introduction

A three-phase induction motor is a widely manufactured electrical machine worldwide with applications in various fields such as cooling systems, chemical, textile, mining, petroleum industries, and especially in electric vehicles (EV) [1]. In recent decades, an Induction Motor Drive (IMD) system applying powerful hardware combined with modern algorithms has overcome the disadvantages of the nonlinear control structure and replaced DC drives in precision control applications, including position, speed, and torque control [2]. There are four main parts in a typical structure of IMDs, including an induction motor connected to a power inverter and a control unit linked with a sensor measurement system. In modern models, the operation of IMDs is a closed-loop control process including the below steps:

- Set a reference value to IMDs corresponding to the specific requirement;
- Supply an initial voltage to start up the motor;
- Measure operational signals by the sensor system and send those feedback signals to the controller;
- The controller conducts the control algorithm and provides the control signal to the power inverter;
- The inverter supplies the corresponding voltage to the motor, then this is repeated.

The performance of IMDs depends on the capacity and stability of all components of the system, where the stable structure of the motor, the analytical model, and the quality of the sensor signals plays an essential role in ensuring the effectiveness of the control process [3]. There is a range of fault diagnosis methods for IMDs, each of which focuses on a specific failure situation, and this research focuses on sensor faults that occur during the operation of IMDs. Operating in harsh environments, sensors of IMDs would possibly be defective due to either mechanical, electrical effects, or chemical corrosion, for example, liquid/solid contaminants, impact, shock, vibration, short circuits, etc. Those such defections could cause either sensor errors or connection failures, leading to the collapse of the entire system. Finding and resolving problems of the sensor errors will enhance the reliability of IMDs; thus, the sensor fault-tolerant control (FTC) methods, integrated into the controller of IMDs against sensor failures [4], have attracted extensive studies in the field of advanced electrical machine control in recent times. Currently, the FTC methods are developed in two main branches: passive fault-tolerant control (PFTC) and active fault-tolerant control (AFTC) [5]. The approaches that are designed to maintain the system’s stable operation under predefined malfunctions without fault detection and reconfiguration functions belong to the PFTC category. Not depending on fault detection and working offline are the advantages of PFTC methods. However, PFTC is only designed against a few simple predefined failures. The system performance relies on redundant hardware structures, which increase the manufacturing and maintenance costs of IMDs [6]. In contrast, AFTC systems use real-time feedback to determine the current operating states of IMDs to apply the appropriate control method without redundant hardware. A typical AFTC system operates in a closed-control loop consisting of three processes: fault detection and diagnosis, failure of signal isolation, and a reconfiguration control strategy. Generally, AFTC systems can resolve various fault types without redundant hardware; however, the effectiveness of AFTC systems depends significantly on the accuracy and decision time of the fault detection and diagnosis phase. Fault diagnosis methods belonging to the AFTC group are reviewed and investigated below.

The field-oriented control (FOC), a typical method belonging to the vector control group, is a modern technique for IMDs. IMDs applying the FOC technique can precisely control torque and rotor flux based on the orthogonal current components in the rotation coordinate system [7,8,9,10]. For operating efficiently, an IMD controlled by the FOC algorithm requires proper feedback signals from sensors, including two current sensors and a speed encoder. The accuracy of these signals plays a significant role in the stability and reliability of the IMD operation. If the failure of these signals is not solved correctly, the IMD may be damaged or collapse. Therefore, integrating the FTC function is necessary to increase the reliability of the IMD system against sensor failures.

The feedback speed from the encoder plays a crucial role in regulating the demand torque for executing the reference speed of IMDs that apply the FOC approach. If the speed sensor signal is faulty, it is necessary to be detected precisely and replaced as quickly as possible. In [11], an extended Kalman filter (EKF) is used as a speed observer to generate a virtual signal parallel to the measured signal in normal operating conditions. A residual value between the virtual signal and measured signal is compared with a defined threshold to detect the fault state of the speed sensor. Similarly, in [12], a current-based MRAS observer is applied for motor speed estimation; the error between the measured signal and the estimated signal is used to diagnose a speed failure. In [13], the average deviation of twenty-speed data points has been used to compare the measured speed and reference speed signals to detect a speed sensor failure. In [14], the authors proposed a method to detect the error of the speed sensor based on the stator flux observer. Approaching the optimization of the IMD system’s operation spread over the whole speed range, in [15], the authors proposed to use two-speed observers, including EKF and Luenbeger Observer (LO), working in parallel with the encoder. The estimated signal of EKF is used in the fault diagnosis algorithm at medium- and low-speed ranges, and the other is used to determine a speed sensor fault at high-speed ranges. Another approach in [16] presented an indirect diagnosis method for detecting a speed sensor failure based on the difference between measured and estimated currents.

The current sensor system of IMDs is classified into two types depending on the control structure: three current sensors and two current sensors. In a typical IMD system using three current sensors, the diagnosis method is based on Kirchhoff’s law principle: the sum of three-phase currents reaches zero at normal states. Three current observers are used to detect sensor failure, and each of them is composed of the corresponding two-phase currents. When a fault occurs with a specified current phase, the two observers corresponding to this phase current will be affected, while the other observer will be unaffected. As a result, the faulty phase will be correctly diagnosed [17,18]. In [19], the LO was used to estimate a virtual space current vector, then the measured and estimated currents were compared with respective phases for each pair in the [a, b, c] coordinate system to detect the current sensor faults. However, if an IMD system is installed with only two current sensors, the principle of Kirchoff’s law is not feasible to detect current sensor faults. In [20], the authors proposed a simple and effective fault current diagnosis method that compares the amplitude of the estimated current in the stationary coordinate system with the amplitude of each measured phase current. However, the fault confirmation time of approximately one cycle is the most significant disadvantage of this method due to its unsuitable application for the low-speed region (long fault detection time). In [21], an asymmetry index created from the subtraction of RMS values of the two-phase currents was used for detecting the current sensor fault state. An axes transformation strategy based on Park transformation has been proposed to detect the current sensor fault [22]. Similarly, another approach that uses a delay function combined with a space vector comparison algorithm in priority order [23] has been proposed to quickly detect current sensor faults for IMDs operating in low-speed ranges. Moreover, a third-difference operator (TDO) in [24,25] has been applied to diagnose a failure in feedback current signals. One advantage of this algorithm is its sensitivity to abnormal changes in measured signals. However, it can cause confusion with random noises, leading to misdiagnosis.

Through the above-reviewed literature, various sensor fault detection (SFD) methods have been classified into five main specified groups, as shown below:

- SFD based on the comparison between the measured signal and the estimated signal, e.g., [11,12,14,15,16,19,20,22];
- SFD based on the comparison between the measured signal and the reference signal, e.g., [13,16];
- SFD based on a self-test with a prior sample signal, e.g., [23,24,25];
- SFD based on the change of a compound signal, e.g., [17,18,21,23];
- SFD based on a mixture of the above-mentioned methods (compound methods), e.g., [16,23].

In this paper, an innovative approach for the current sensor fault diagnosis based on the rotor slip estimation combined with the TDO technique is proposed to precisely locate a faulty phase current according to a predefined priority. It belongs to the compound methods, where the quality of both the current measurement and the speed encoder are verified and estimated simultaneously. Most existing sensor fault diagnosis methods do not mention random noises on feedback signals, which can lead to a mismatch diagnosis during the operation of IMDs. Therefore, the innovative standout of the proposed method from previous studies is the ability to distinguish between certain failures and random noises of the current sensors to make precise diagnosis decisions for increasing reliability in the control process. In the diagnosis method, the initial indication of a current sensor failure is first recorded by the TDO technique and then verified by the rotor slip comparison algorithm. The speed sensor fault diagnosis algorithm will be next performed as soon as the health status of the current sensors is verified. Therefore, by combining a reasonable diagnosis sequence, the proposed method has a faster sensor fault detection time than other diagnosis methods based on RMS values while ensuring high accuracy. Once the sensor fault is identified, the faulty measured signal will be replaced by an appropriate estimated signal to ensure the stable operation of the IMD. Moreover, most of IMD’s controllers achieve good performance when operating close to the nominal speed; however, they could face various difficulties at the low-speed region due to the influence of varying machine parameters, inverter nonlinearities, and a longer processing time [26]. This paper demonstrates the effectiveness of the proposed approach under sensor fault conditions in a low-speed zone. Many simulations and experiments have been conducted to prove the feasibility of the proposed technique.

The article is structured in five sections. The first part presents the introduction to research problems, and the second describes a mathematical description of the FOC and some sensor fault types. The following two parts, respectively, offer the simulation and experimental results in a low-speed range to prove the technical feasibility. The contributions and discussion of this research are described in the last section.

## 2. Mathematical Modeling of the Induction Motor

Unlike DC motors, the relationships of induction motor (IM) electromagnetic quantities are complex and nonlinear. In the [α, β] stationary reference frame, the dynamic modeling of the IM can be described with the differential form [17], as below:

(1) $$\left\{ \begin{array}{l} \frac{di_{S\alpha }}{dt}=-Ai_{S\alpha }+B\psi_{R\alpha }+Cp\omega \psi_{R\beta }+Du_{S\alpha }\\ \frac{di_{S\beta }}{dt}=-Ai_{S\beta }+B\psi_{R\beta }-Cp\omega \psi_{R\alpha }+Du_{S\beta } \end{array}\right.,$$

(2) $$\left\{ \begin{array}{l} \frac{d\psi_{R\alpha }}{dt}=Ei_{S\alpha }-F\psi_{R\alpha }-p\omega \psi_{R\beta }\\ \frac{d\psi_{R\beta }}{dt}=Ei_{S\beta }-F\psi_{R\beta }-p\omega \psi_{R\alpha } \end{array}\right.,$$

(3) $$T_{e}=\frac{3p}{2}\frac{L_{m}}{L_{R}}\left(\psi_{R\alpha }i_{S\beta }-\psi_{R\beta }i_{S\alpha }\right),$$

where:

$\begin{array}{l} A=\frac{R_{S}L_{R}^{2}+R_{R}L_{m}^{2}}{\sigma L_{S}L_{R}^{2}};B=\frac{L_{m}R_{R}}{\sigma L_{S}L_{R}^{2}};C=\frac{L_{m}}{\sigma L_{S}L_{R}};D=\frac{1}{\sigma L_{S}};E=\frac{L_{m}R_{R}}{L_{R}};F=\frac{R_{R}}{L_{R}};\sigma =(1-\frac{L_{m}^{2}}{L_{S}L_{R}}) \end{array}$

$\begin{array}{l} T_{e}:\mathrm{Electrical}\;\mathrm{torque};\\ i_{S\alpha }/i_{S\beta }:\mathrm{Stator}\;\mathrm{current}\;\mathrm{components}\;\mathrm{in}\;\left[\alpha \;\beta \right];\\ u_{S\alpha }/u_{S\beta }:\mathrm{Stator}\;\mathrm{voltage}\;\mathrm{components}\;\mathrm{in}\;\left[\alpha \;\beta \right];\\ \psi_{R\alpha }/\psi_{R\beta }:\mathrm{Rotor}\;\mathrm{flux}\;\mathrm{components}\;\mathrm{in}\;\left[\alpha \;\beta \right];\\ p:\mathrm{Pole}\;\mathrm{pair}\;\mathrm{number};\\ R_{S}/R_{R}:\mathrm{Stator}/\mathrm{Rotor}\;\mathrm{resistance};\\ L_{S}/L_{R}/L_{m}:\mathrm{Stator}/\mathrm{Rotor}/\mathrm{Magnetizing}\;\mathrm{inductance}; \end{array}$

**Figure 1:**
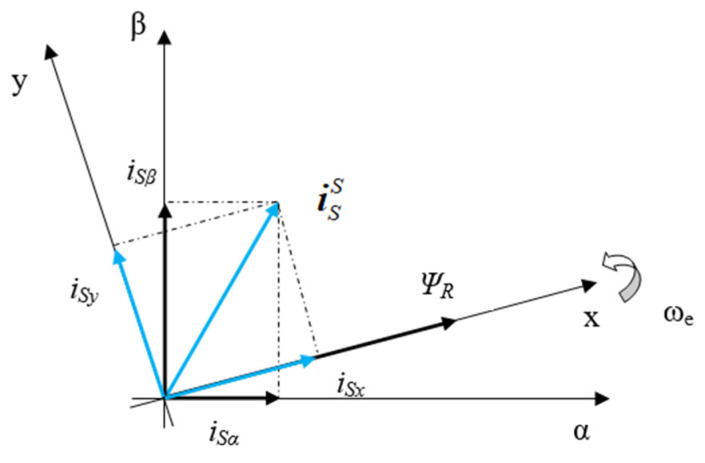
Current space vector decomposition.

**Figure 2:**
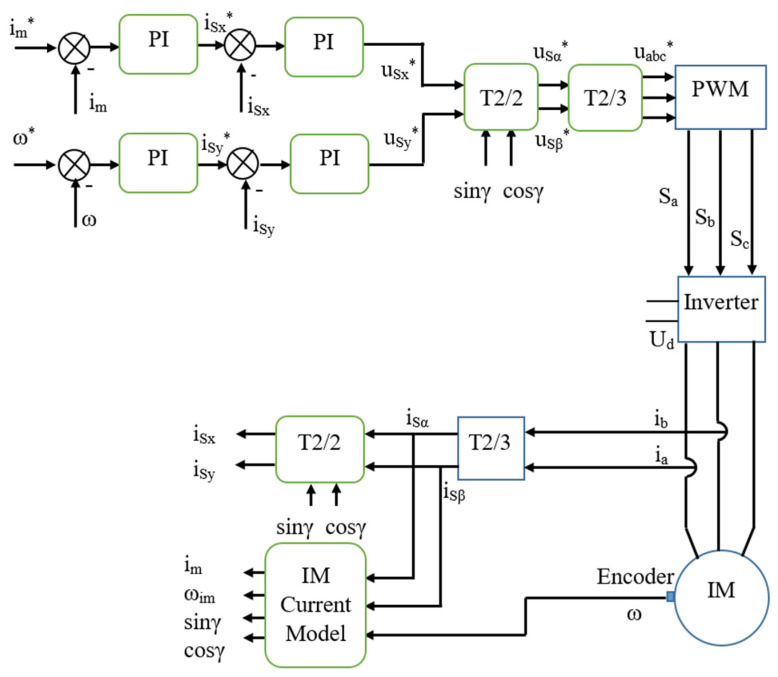
Block diagram of IMD with the FOC strategy (the symbol * means reference values).

In order to satisfy the requirement of independent control of flux and torque, the FOC method is often used in high-performance variable-speed drives. In the FOC method, the complexity of the motor control model is linearized by decomposing the current space vector into two perpendicular components in the [x, y] rotating coordinate system. It rotates with the same angular velocity as the rotor flux vector and x-axis corresponds to the rotor flux, as shown in Figure 1. A typical FOC-based control system includes controllers, a voltage source inverter, an AC motor, and sensors, as shown in Figure 2. Control demands such as the reference rotor flux or desired speed are set as the inputs for the control algorithm, which generates switching pulses for the inverter. The inverter provides energy to the IM in accordance with the control commands. Furthermore, during an operation, the measured signals from the sensors are fed back to the control loops.

In the FOC loops, the two measured currents are converted to the [α, β] stationary coordinate system and [x, y] rotating coordinate system by Clarke’s and Park’s formulas, as shown below:

(4) $$\left[\begin{array}{l} i_{S\alpha }\\ i_{S\beta } \end{array}\right]=\left[\begin{array}{l} \;1\;\;\;\;\;\;\;\;\;\;\;0\\ \frac{1}{\sqrt{3}}\;\;\;\;\;\;\;\frac{2}{\sqrt{3}} \end{array}\right]\times \left[\begin{array}{l} i_{a}\\ i_{b} \end{array}\right],$$

(5) $$\left[\begin{array}{l} i_{Sx}\\ i_{Sy} \end{array}\right]=\left[\begin{array}{l} \;\;\;\cos\gamma \;\;\;\;\;\sin\gamma \\ -\sin\gamma \;\;\;\;\;\cos\gamma \end{array}\right]\times \left[\begin{array}{l} i_{S\alpha }\\ i_{S\beta } \end{array}\right],$$

In the [x, y] frame, the dynamic modeling of the IM can be described by the following differential expressions:

(6) $$\mathrm{Stator}:\left\{ \begin{array}{l} u_{Sx}=R_{S}i_{Sx}+\frac{d\psi_{Sx}}{dt}-\omega_{im}\psi_{Sy}\\ u_{Sy}=R_{S}i_{Sy}+\frac{d\psi_{Sy}}{dt}+\omega_{im}\psi_{Sx} \end{array}\right.,$$

(7) $$\mathrm{Rotor}:\left\{ \begin{array}{l} 0=R_{R}i_{Rx}+\frac{d\psi_{Rx}}{dt}-\omega_{sl}\psi_{Ry}\\ 0=R_{R}i_{Ry}+\frac{d\psi_{Ry}}{dt}+\omega_{sl}\psi_{Rx} \end{array}\right.,$$

(8) $$\mathrm{Stator}\;\mathrm{flux}:\left\{ \begin{array}{l} \psi_{Sx}=L_{S}i_{Sx}+L_{m}i_{Rx}\\ \psi_{Sy}=L_{S}i_{Sy}+L_{m}i_{Ry} \end{array}\right.,$$

(9) $$\mathrm{Rotor}\;\mathrm{flux}:\left\{ \begin{array}{l} \psi_{Rx}=L_{R}i_{Rx}+L_{m}i_{Sx}\\ \psi_{Ry}=L_{R}i_{Ry}+L_{m}i_{Sy} \end{array}\right.,$$

where:

$\begin{array}{l} i_{Sx}/i_{Sy}:\mathrm{Stator}\;\mathrm{current}\;\mathrm{components}\;\mathrm{in}\;\left[x\;y\right];\\ i_{Rx}/i_{Ry}:\mathrm{Rotor}\;\mathrm{current}\;\mathrm{components}\;\mathrm{in}\;\left[x\;y\right];\\ u_{Sx}/u_{Sy}:\mathrm{Stator}\;\mathrm{voltage}\;\mathrm{components}\;\mathrm{in}\;\left[x\;y\right];\\ \psi_{Sx}/\psi_{Sy}:\mathrm{Stator}\;\mathrm{flux}\;\mathrm{components}\;\mathrm{in}\;\left[x\;y\right];\\ \psi_{Rx}/\psi_{Ry}:\mathrm{Rotor}\;\mathrm{flux}\;\mathrm{components}\;\mathrm{in}\;\left[x\;y\right];\\ \omega_{im}:\mathrm{Flux}\;\mathrm{angular}\;\mathrm{speed};\\ \omega_{sl}:\mathrm{Electrical}\;\mathrm{rotor}\;\mathrm{slip}\;\mathrm{speed}; \end{array}$

In the normal operating condition, the rotor flux, according to the FOC method, is fixed as a constant value and $i_{Sy}$ is the only component to regulate the motor speed. Therefore, the IM’s rotor flux can be rewritten by:

(10) $$\left\{ \begin{array}{l} \psi_{Rx}=\psi_{R}=const\\ \psi_{Ry}=0 \end{array}\right.,$$

By applying (10) to the dynamic modeling, we can obtain the characteristic control equations in the [x, y] coordinate system [27], as below:

(11) $$\left\{ \begin{array}{l} u_{Sx}=R_{S}i_{Sx}-\omega_{im}L_{\sigma }i_{Sy}\\ u_{Sy}=R_{S}i_{Sy}+\omega_{im}L_{S}i_{Sx}+L_{\sigma }\frac{di_{Sy}}{dt} \end{array}\right.,$$

From (10) and (11), the stator current components can be determined by the following equations:

(12) $$\left\{ \begin{array}{l} \frac{di_{Sy}}{dt}=\frac{1}{L_{\sigma }}\left[-R_{S}i_{Sy}-L_{S}\omega_{im}i_{Sx}+u_{Sy}\right]\\ i_{Sx}=\frac{\omega_{im}L_{\sigma }i_{Sy}+u_{Sx}}{R_{S}} \end{array}\right.,$$

(13) $$\left\{ \begin{array}{l} \omega_{im}=p\omega +\omega_{sl}\\ \psi_{R}=L_{m}i_{m} \end{array}\right.,$$

where:

$\begin{array}{l} L_{\sigma }=(\frac{L_{S}L_{R}-L_{m}^{2}}{L_{R}});\\ \omega :\mathrm{Mechanical}\;\mathrm{angular}\;\mathrm{speed};\\ i_{m}:\mathrm{Magnetizing}\;\mathrm{current}; \end{array}$

The electrical rotor slip speed can be determined as (14):

(14) $$\omega_{sl}=\frac{i_{Sy}}{T_{R}i_{m}},$$

where:

$T_{R}:\mathrm{Rotor}\;\mathrm{time}\;\mathrm{constant};$

Generally, the accuracy of the feedback-measured signals, such as the stator currents and rotor speed, plays a vital role in the performance of control systems based on the machine model. If the quality of the feedback signals is inappropriate, the control algorithms will not be implemented correctly, leading to low control effectiveness or, worse, the collapse of the system completely. Abnormal situations of sensors can occur in various forms, which can be classified into two main types: partial failure and hard failure (or called total sensor fault) [28,29,30]. Partial failures, such as drift, scaling, bias, etc., often occur due to the sensor’s quality deterioration. Such problems are uncertain and very difficult to detect by observing the signal during operation. Otherwise, such as most severe sensor failures, hard failure (or complete faults) often occurs instantaneously and makes endangered IMDs. This paper focuses on diagnosing the type of total faults in which sensors would be damaged entirely and feedback signals reach zero, causing severe problems for the IMD operation. Hence, the reliable determination and quick isolation of any occurred faults are the essential requirements of fault diagnosing methods. Moreover, power supply units based on the PWM technique can generate a random pulse noise to the current signals [31,32]. It can also occur in the speed signal from the encoder by a bit-error of the data reading process. These noises are less severe, appear only for a short time, and can be handled by filters in the control algorithm. Therefore, the fault diagnosis algorithm must ignore these noises and not affect the regular operation of IMDs.

## 3. Sensor Fault Diagnosis Method Based on Rotor Slip

### 3.1. FTC Function Using the Typical FOC Strategy

**Figure 3:**
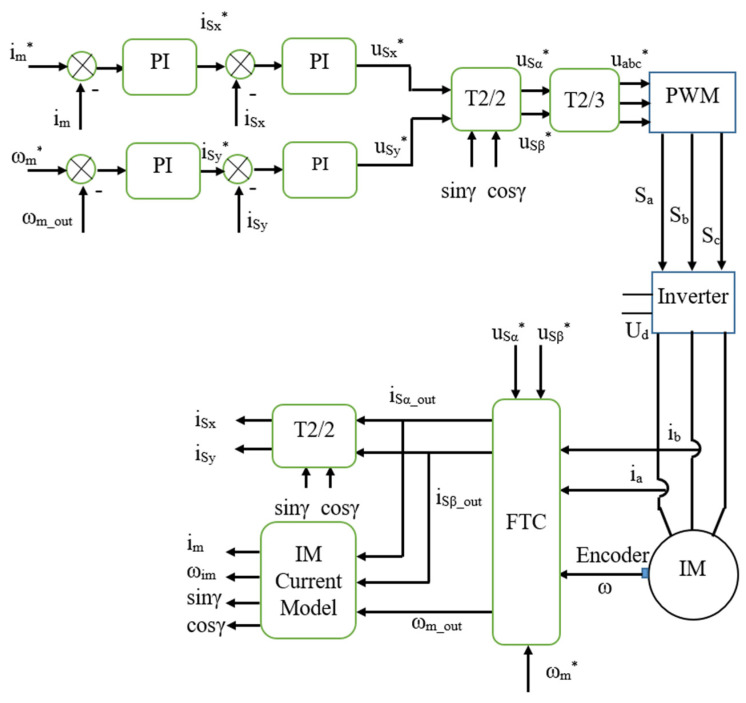
Block diagram of FOC-based IMD using FTC functions (the symbol * means reference values).

As shown in Figure 3 below, the FOC of IMDs needs at least two current sensors and an encoder to provide the feedback signals. Additionally, an FTC function is installed in the control system to diagnose the operating status and provide the appropriate signal to the control algorithms.

The FTC outputs include two groups, status indicators (FA_Flag, FB_Flag, FW_Flag) and feedback values ($i_{S\alpha \_out},\;i_{S\beta \_out},\;\omega_{m\_out}$), which are provided to the control loops of the FOC. Suppose that the IMD system is operating in normal conditions, and the outputs of the FTC are the measured rotor speed and the [α, β] components of the stator current vector—if any single sensor fault happens in either the speed sensor or current sensor, the corresponding estimated quantities will replace such faulty sensor signals at the FTC outputs to keep IMDs in a stable operation. At the same time, the corresponding sensor flag will also indicate the exact error type.

**Figure 4:**
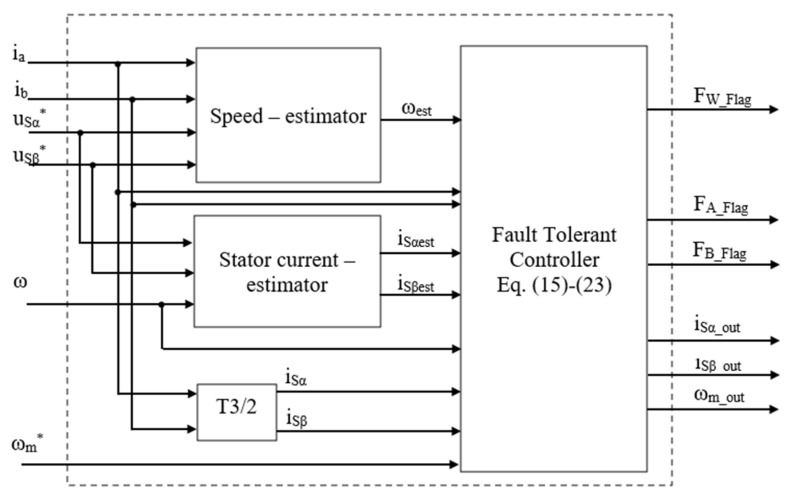
Block diagram of FTC unit (the symbol * means reference values).

Figure 4 presents the FTC function unit, and ($u_{S\alpha^{*}},\;u_{S\beta^{*}},\;i_{a},\;i_{b},\;\omega ,\;\omega_{m^{*}}$) are inputs which include the reference voltage signals, measured currents, measured rotor speed, and reference speed. The three-phase currents obtained from the sensors are transformed into the [α, β] stationary coordinate system in block T3/2 by (4). The speed and stator current estimators use proper algorithms to generate the estimated quantities for the diagnostic and compensation processes. Methods based on the typical machine model, such as MRAS techniques, can be applied to estimate the virtual rotor speed [33,34,35,36]. Furthermore, the sliding mode (SMO) method [37,38,39,40], which is less affected by external noise and the accuracy of machine parameters, is also a practical choice for speed estimation [40] in this strategy. On the other hand, the proper algorithms can be applied to the stator current estimator to generate the virtual currents $\left(i_{S\alpha est},\;i_{S\beta est}\right)$ provided to the FOC control loop [41,42,43]. The Luenberger observer-based current estimator [43], which has a fast, dynamic performance corresponding with various operating conditions, is applied to estimate the virtual currents for the proposed diagnosis.

The conventional sensor fault diagnosis methods rely on the difference between the measured and estimated values to determine the fault conditions. In this approach, a virtual parameter, i.e., the stator current or rotor speed, can usually be calculated from the mathematical model as mentioned above and the other measured quantity. However, it is highly effective when only a single failure occurs in either the speed sensor or the current sensor. If a double failure of the speed and current sensors occurs, the conventional diagnosis functions perform ineffectively.

### 3.2. Advanced Diagnosis Method for Current Sensors Based on Rotor Slip

This paper proposes an effective real-time diagnosis strategy to reliably detect any failure for both the speed and current sensors. Moreover, to enhance the reliability of the fault diagnosis of the current sensors, a rotor slip (RSL)-based comparison algorithm is proposed to cancel random noises in the current measurement system. The method uses the reference rotor slip determined from the reference components of the stator current vector and rotor time constant using the current model. The model inputs consist of the measured currents “$i_{S\alpha },\;i_{S\beta }$” and reference speed “$\omega_{m^{*}}$”. The RSL is used as a criterion to distinguish between the failure and noise of the current sensors. The mathematical expressions to determine the reference rotor slip [9] can be described below:

(15) $$\left[\begin{array}{l} i_{Sd}^{*}\\ i_{Sq}^{*} \end{array}\right]=\left[\begin{array}{l} \;\;\;\;\cos\varepsilon^{*}\;\;\;\;\;\sin\varepsilon^{*}\;\;\\ -\sin\varepsilon^{*}\;\;\;\;\;\;\cos\varepsilon^{*}\; \end{array}\right]\left[\begin{array}{l} i_{S\alpha }\\ i_{S\beta } \end{array}\right],$$

where $\varepsilon^{*}=\int p\omega_{m}^{*}dt;$

(16) $$\left\{ \begin{array}{l} i_{md}^{*}=\frac{1}{T_{R}s+1}i_{Sd}^{*}\\ i_{mq}^{*}=\frac{1}{T_{R}s+1}i_{Sq}^{*} \end{array}\right.,$$

(17) $$\left[\begin{array}{l} i_{m\alpha }^{*}\\ i_{m\beta }^{*} \end{array}\right]=\left[\begin{array}{l} \cos\varepsilon^{*}\;\;\;\;\;-\sin\varepsilon^{*}\;\;\\ \sin\varepsilon^{*}\;\;\;\;\;\;\;\;\;\cos\varepsilon^{*}\; \end{array}\right]\left[\begin{array}{l} i_{md}^{*}\\ i_{mq}^{*} \end{array}\right],$$

(18) $$\mathrm{Rotor}\;\mathrm{flux}\;\mathrm{angle}:\;\gamma^{*}=arctg(\frac{i_{m\beta }^{*}}{i_{m\alpha }^{*}}),$$

Applying (5) and (14) with $\gamma^{*}$, we can obtain the reference rotor slip as:

(19) $$\omega_{sl}^{*}=\frac{i_{Sy}^{*}}{T_{R}i_{Sx}^{*}},$$

In a normal operation, as presented by (19), the reference RSL, $\omega_{sl^{*}}$, is a parameter depending only on the measured currents and not on the rotor speed. If a random noise pulse occurs, the reference RSL fluctuates in a short time only and quickly returns to its original state. However, if a certain failure occurs in any current sensor, the reference RSL will periodically strongly oscillate. Therefore, the fault diagnosis algorithm of the current sensors using the reference RSL can reliably recognize a certain failure of the current sensors, but is unable to detect exactly the location of the damaged phase current sensor.

### 3.3. Proposed General Diagnosis Strategy for Current and Speed Sensors

**Figure 5:**
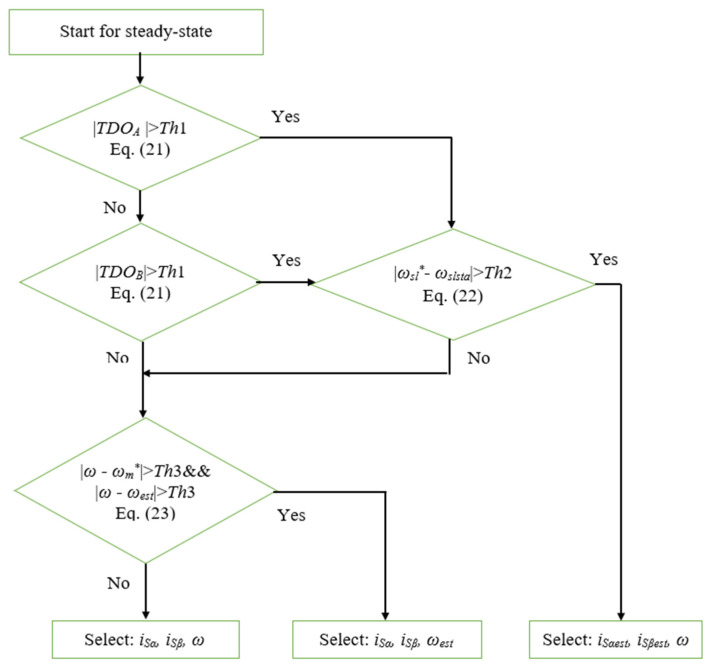
Block diagram of the FTC unit (the symbol * means reference values).

This paper proposes an effective diagnosis strategy for both the current and speed sensors. The diagnosis algorithm proceeds with sequential checking steps, starting from the current sensor status and then the speed sensor condition. The flowchart presenting the proposed general fault diagnosis algorithm is described in Figure 5.

As we can see in the flowchart, the current sensor diagnosis is first executed by two sub-steps, TDO and reference RSL. The TDO is used to determine any change in a specific phase current, and the reference RSL confirms a certain fault of the current sensors in the current measurement. The TDO method-based current fault detection algorithm, including three difference operators [24,25,44], is implemented to recognize a fault of phase currents in real-time. Those difference operators compare the stator current values of the same specific phase at two different sampling periods, the second-order and third-order differences of the current change. The operators of the TDO technique are described by three expressions below:

(20) $$\left\{ \begin{array}{l} 3^{rd}\;\mathrm{difference}\;\mathrm{operator}:\;\Delta^{3}i_{j}(n)=\Delta^{2}i_{j}(n)-\Delta^{2}i_{j}(n-1)\\ 2^{nd}\;\mathrm{difference}\;\mathrm{operator}:\;\Delta^{2}i_{j}(n)=\Delta^{1}i_{j}(n)-\Delta^{1}i_{j}(n-1)\\ 1^{st}\;\mathrm{difference}\;\mathrm{operator}:\;\Delta^{1}i_{j}(n)=i_{j}(n)-i_{j}(n-1) \end{array}\right.,$$

where j is the phase of the current sensor; n is the current sampling time; n−1 is the previous sampling time.

In normal operating conditions, the difference between the phase current values at two sampling times is very small, and the operators of the TDO method are also limited to a small range. However, if any sudden change in the phase current occurs due to either a noise or sensor fault, the indexes will significantly increase. Hence, in order to recognize a phase current change, the proposed current diagnosis algorithm applies the checking criterion based on the third difference operator of the stator currents defined in (21):

(21) $$\left\{ \begin{array}{l} TDO_{j}=\left|\Delta^{3}i_{j}(n)\right|;\\ If(TDO_{j}>=Th1)\{F_{TDOj}=1;\} \end{array}\right.,$$

The threshold $Th_{1}$ should be selected higher than the values of the $TDO_{j}$ index under normal operating conditions. Based on the reference [24] recommendations and our extensive experimental testing, we set $Th_{1}$ with a value of 0.5 A.

**Figure 6:**
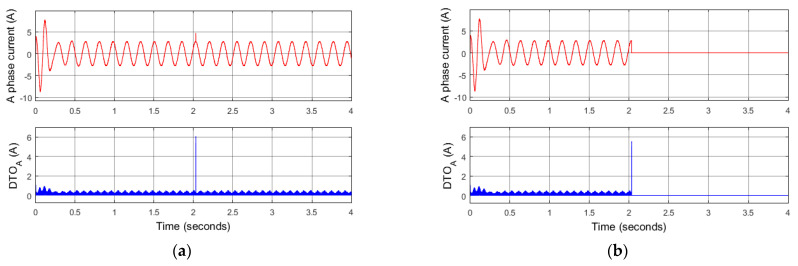
Simulation results: changes of TDO indexes in cases: (**a**) random noise of A phase current signal; (**b**) total fault of A phase current sensor.

Although the TDO technique is very sensitive to detecting changes in the current signal, it is not able to distinguish between permanent fault states and random noise. For example, when one pulse noise occurs at the time of 2 s in Figure 6a, the corresponding TDO index indicates the same result as the TDO index of the occurred current sensor fault in Figure 6b.

**Figure 7:**
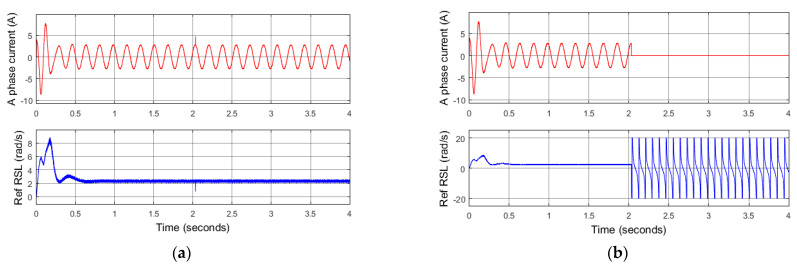
Simulation results: changes of reference RSL signal in cases. (**a**) Random noise of A phase current signal; (**b**) total fault of A phase current sensor.

The reference RSL calculated by (19) is then applied to confirm a certain fault of the current sensors, not random noise in the current measurement. As shown in Figure 7a, when a random noise pulse occurs, the reference RSL only fluctuates in a short time and quickly returns to its original state. However, if a real failure occurs in any current sensor, the reference of RLS will periodically strongly oscillate, as shown in Figure 7b.

By applying the RSL technique, the fault diagnosis algorithm to determine the current sensor faults can be modified, as shown below:

(22) $$\begin{array}{l} If(\omega_{sl}^{*}-\omega_{slsta}>=Th2)\\ \left\{Cur\_counter=Cur\_counter+1;\right\}\\ Else\;\{Cur\_counter=0;\}\\ If(Cur\_counter>Cur\_Coe)\\ \left\{If(F_{TDOA}=1)\{F_{A\_Flag}=1;\right\}\\ If(F_{TDOB}=1)\{F_{B\_Flag}=1;\}\} \end{array},$$

where $\omega_{slsta}$ is the mean value of the rotor slip corresponding to a current period in the steady-state operation, Cur_coe is a set time of a current counter used to avoid random pulse noises. In this paper, the hardware of the practical experiments uses DSC-TMS320F28335 to implement the control algorithm, and each processing cycle of the DSC is set to a value of 100 µs. To ensure the reliable and stable operation of the IMD system, Cur_coe should be set at 20 sampling cycles, which is equal to a current fault determination time of 2 ms. This value of Cur_coe is suitable because most of the random pulse noises occur in intervals of less than 2 ms. Additionally, the threshold $Th_{2}$ serves as a limit difference between the actual recent slip and its steady value. According to practical experience through various experiments, the value of 10% of the reference rotor speed should be set for $Th_{2}$ to make reliable results. It implies that the defective sensor signal should be verified by not only the value deviation but also the existence duration.

Remark: the value of $\omega_{slsta}$ will affect the accuracy of the RLS diagnosis technique, therefore, $\omega_{slsta}$ needs to be updated every current cycle to avoid the effects of load changes during the operation.

As we can see in the flowchart, after checking the current sensor status, the diagnostic procedure for the speed sensor is then performed by comparing the reference and estimated speed with the measured rotor speed combined with a counter as a time-checker. It implies that the defective speed sensor signal should be verified by not only the value deviation but also the existence duration. The implementation can be described by the following steps:

(23) $$\begin{array}{l} If(((\omega -\omega_{m}^{*})>=Th3)\&\&((\omega -\omega_{est})>=Th3))\\ \left\{Sp\_counter=Sp\_counter+1;\right\}\\ Else\{Sp\_counter=0;\}\\ If(Sp\_counter>Sp\_Coe)\\ \left\{F_{W\_Flag}=1;\right\} \end{array},$$

$Th_{3}$ is a limitation for detecting the speed sensor fault. It depends on the range of the motor speeds [14] and can be implemented by:

$$Th3=\left\{ \begin{array}{l} 0.045*\left|\omega_{m}^{*}\right|;\;if\;\left|\omega_{m}^{*}\right|>200\;\mathrm{rpm}\\ 0.1*\left|\omega_{m}^{*}\right|;\;if\;\left|\omega_{m}^{*}\right|\leq 200\;\mathrm{rpm} \end{array}\right.,$$

Sp_coe is a set time of a speed counter used to avoid random pulse noises. According to practical experience through various experiments, the Sp_coe should be set in a range of 30–40 sampling cycles (3 to 4 ms) to obtain a fast and reliable detection and avoid a mismatch due to random noises.

Finally, the proposed FTC algorithm consists of various diagnosis blocks, each of which detects a specific type of sensor fault. Table 1 presents the output flags of sensor faults and the corresponding states.

## 4. Simulation Results

The simulation model of the IMD is built using the nominal motor parameters (see Table 2). The IMD based on the FOC control structure is presented in Figure 3. The model integrates the FTC function with the measurement system consisting of two current sensors and the speed encoder. Each total sensor failure was simulated to verify the performance of the proposed fault diagnosis method during the IMD operation.

The control algorithms usually achieve high efficiency in the rated speed region because of the stability of the machine parameters. In contrast, control algorithms often reduce their efficiency when the IMD system works in the low-speed area due to changes in the motor parameters and the nonlinearity of the power converter. Therefore, to demonstrate the effectiveness of the proposed algorithm, simulations have been conducted to verify the capability to detect various sensor failures at 10% of the rated motor speed.

**Figure 8:**
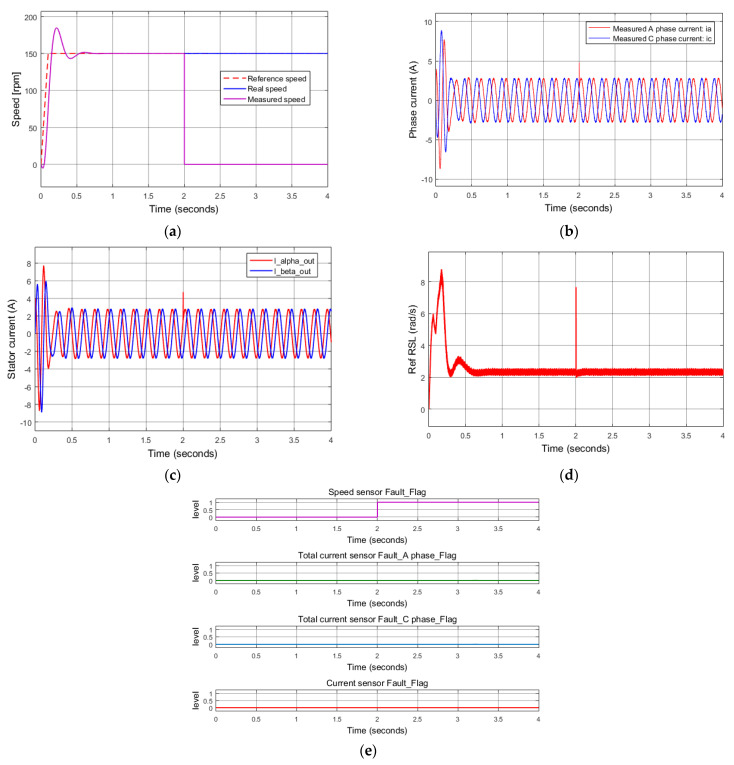
Simulation results: total rotor speed sensor failure at 2 s. (**a**) Reference, real, measured speeds; (**b**) measured current; (**c**) stator current vector components for FOC controllers (FTC outputs); (**d**) reference rotor slip; (**e**) sensor fault indications.

First, the induction motor operates with all healthy sensors, then sudden damage to the speed sensor occurs at 2 s, as shown in Figure 8a. Thus, the feedback sensor signal is disconnected, and the controller receives a speed value of zero. At this moment, a speed sensor failure is detected due to the difference between the reference rotor speed and the sensor feedback signal (23). This problem is quickly recognized just after 35 sampling cycles (3.5 ms). Moreover, as we can see in Figure 8b–d, at the time of 2 s, there is a noise in the stator phase current affected by the damage of the speed sensor, so the current diagnosis function based on the RSL algorithm is also initiated. The current diagnosis function correctly recognizes no failure of the current sensors within 20 sampling cycles (2 ms) and quickly releases (no fault code issued). We can see in Figure 8d that the signal of the reference RSL surged at the time of 2 s and came back to the normal state thereafter. Finally, only the speed sensor flag is activated in Figure 8e, which depicts the results of the FTC (Table 1), where each indication error flag specifies the corresponding error of the used sensors.

**Figure 9:**
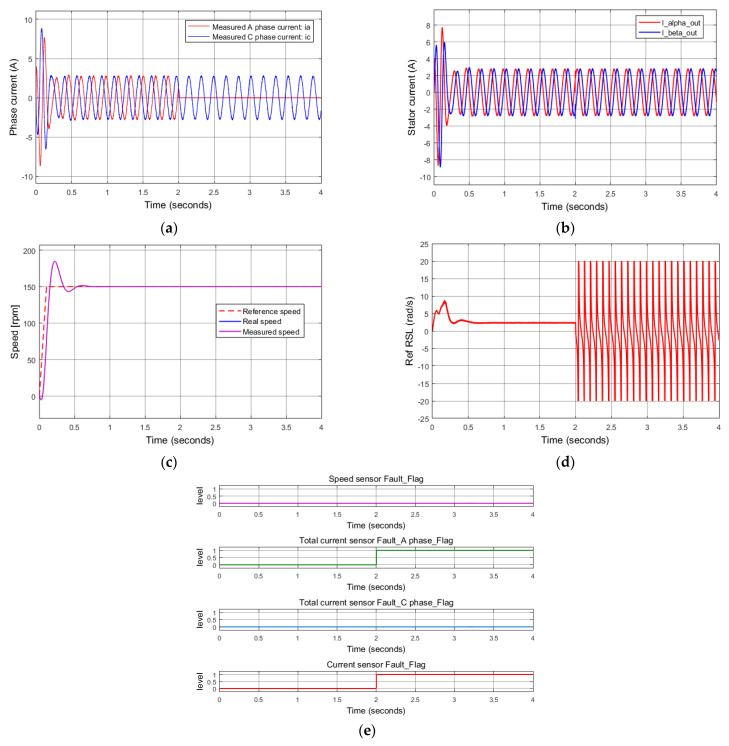
Simulation results: Total A phase current sensor failure at 2 s. (**a**) Measured currents; (**b**) stator current vector components for FOC controllers (FTC outputs); (**c**) reference, real, measured speeds; (**d**) reference rotor slip; (**e**) sensor fault indications.

The second simulation describes a disconnected failure of current sensors at phase A. As shown in Figure 9a, at the time of 2 s, the current signal of phase A is lost. The TDO-based current diagnosis algorithm immediately recognizes an abnormal state of the current sensor of phase A. Then, the current diagnosis algorithm based on the reference RSL is activated to confirm a certain fault of the current sensors. The execution of two diagnosis algorithms can be seen in Equations (21) and (22) of the previous section. In this case, the critical current sensor fault is quickly determined within 20 sampling cycles (2 ms) only. Figure 9d presents the signal of the reference RSL, which contains periodical oscillations after losing the phase A current signal. Finally, Figure 9e depicts the results of the FTC unit, where two indication error flags corresponding to the exact phase failure (phase A) and the fault type (current sensor or other) are activated simultaneously.

**Figure 10:**
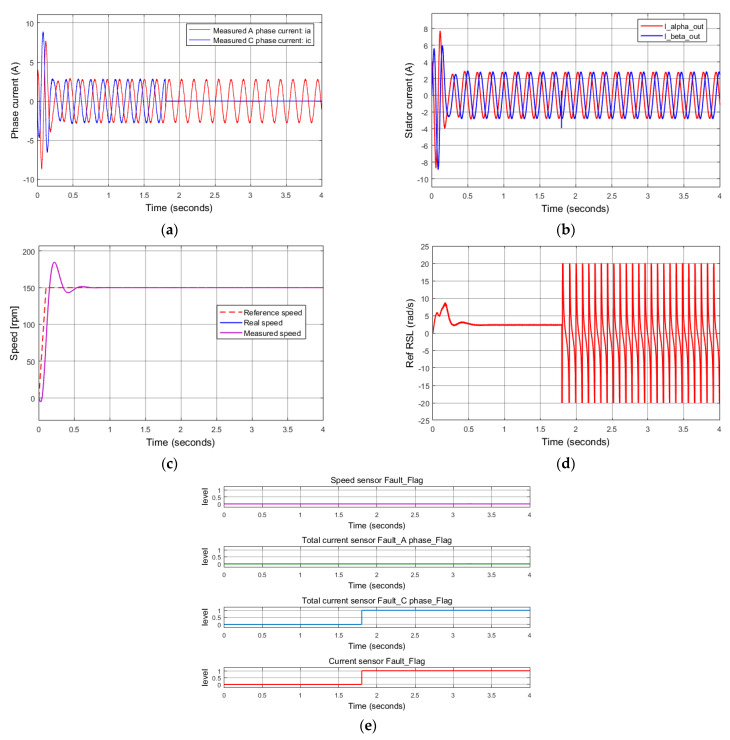
Simulation results: total C phase current sensor failure at 1.8 s. (**a**) Measured currents; (**b**) stator current vector components for FOC controllers (FTC outputs); (**c**) reference, real, measured speeds; (**d**) reference rotor slip; (**e**) sensor fault indications.

Similarly, if there is a disconnection of the current sensor at phase C, the diagnosis algorithm can also determine exactly the phase damaged. Figure 10 describes the performance of the last simulation, mentioning the situation of the current sensor damaged at phase C. It looks very similar to the responses of the situation of the current sensor disconnected at phase A.

After these three simulation cases, it can be stated that the proposed diagnosis algorithm has proved its effectiveness in diagnosing various sensor failures at the low-speed range of induction motors. It has responded very fast and reliably to the damage states of both the speed and current sensors. The problem of the speed sensor is quickly resolved within 35 sampling cycles (3.5 ms), and the problem of the current sensors is certainly detected even faster, with only 20 sampling cycles (2.0 ms). It could help the IMD system switch from the normal to the fault-tolerant modes very smoothly due to the prompt and precise reaction of the proposed FTC method. During the fault-tolerant operation, the estimated quantities are used instead of the corresponding measured signals.

## 5. Experimental Results

**Figure 11:**
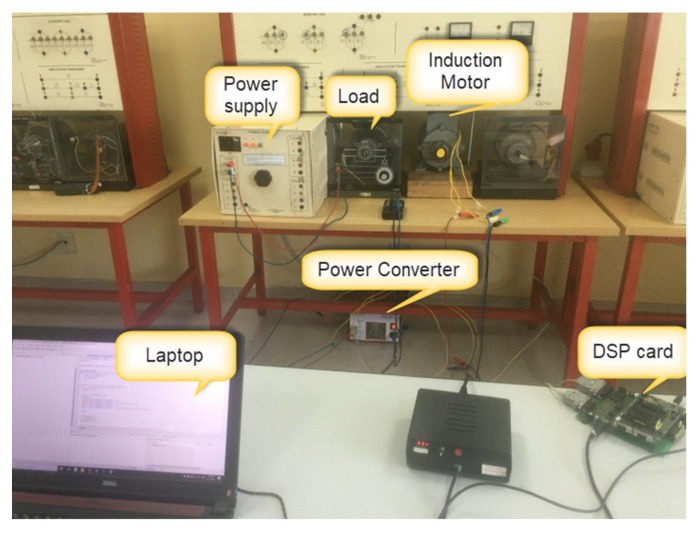
Experimental IMD model.

To evaluate the ability of the proposed FTC method in practice, many practical experiments have been implemented by a real IMD system, as shown in Figure 11, which includes an induction motor, a variable load, a power inverter unit controlled by the DSC TMS320F28335, and a three-phase power supply. The control algorithms (application software) are developed using the C++ programming language in the Code Composer Studio (CCS) programming environment.

In order to demonstrate the practical capability of the proposed approach, the experimental scenarios have been set up the same as the simulations mentioned in the previous section. The real induction motor drive parameters correspond to the motor parameters used for the simulation (see Table 2). The experiments have verified three sensor fault types: total speed sensor fault and total current sensor failures of phases A and C at the low-speed region where the operating motor speed is set to 150 rpm (10% of rated motor speed) to investigate the correlation between the simulation and practical results.

**Figure 12:**
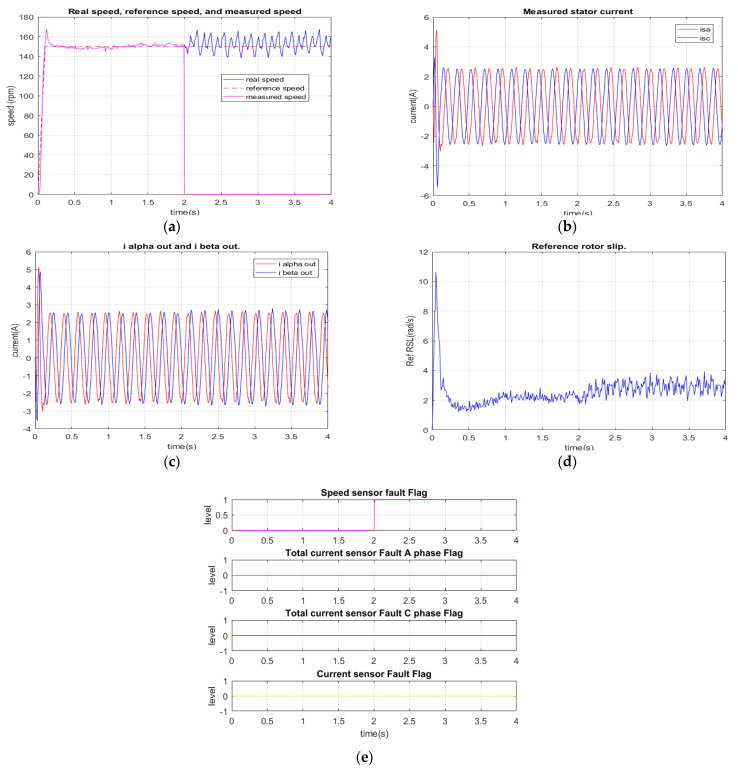
Experimental results: total rotor speed sensor failure at 2 s. (**a**) Reference, real, measured speeds; (**b**) measured current; (**c**) stator current vector components for FOC controllers (FTC outputs); (**d**) reference rotor slip; (**e**) sensor fault indications.

In the first experiment, the induction motor drive operates in normal conditions with all healthy sensors from 0 to 2 s, as shown in Figure 12a. However, a sudden speed sensor damage occurs at the time of 2 s, so the feedback speed signal reaches a value of zero. At this moment, the speed sensor diagnosis algorithm is activated due to a sufficient difference between the reference value and the feedback signal during 35 sampling cycles. As we can see in Figure 12b–d, current sensors are in good condition, and the reference RSL is in a stable state (with a small variation). Thus, except for the speed sensor flag, other fault indicators corresponding to other sensor types still remain zero, as described in Figure 12e. The execution time of the diagnosis algorithm takes 3.5 ms, corresponding to 35 processing cycles of TMS320F28335, which is 100 µs/cycle. Additionally, this experiment result is similar to the relevant simulation result.

**Figure 13:**
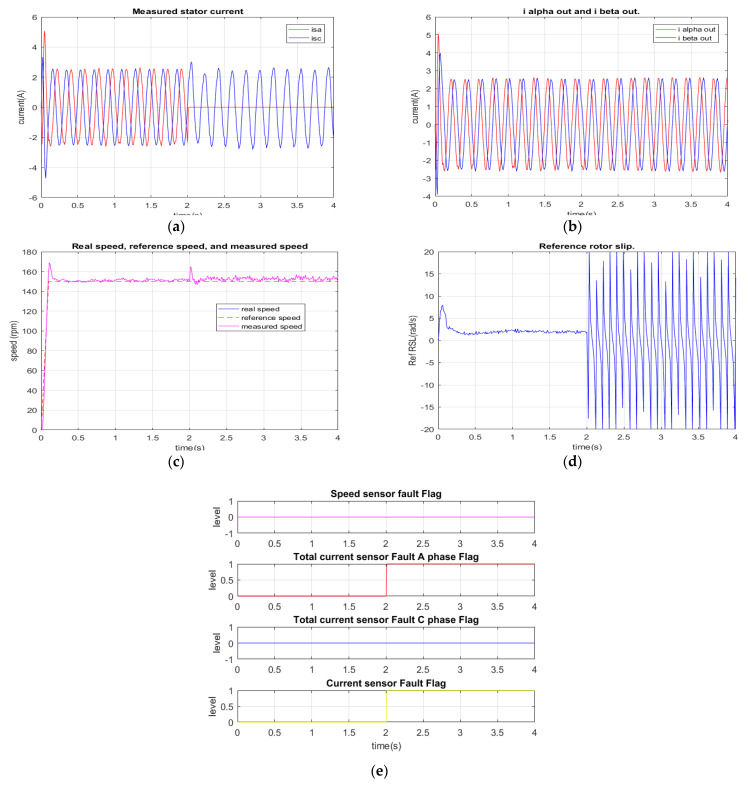
Experimental results: total A phase current sensor failure at 2 s. (**a**) Measured currents; (**b**) stator current vector components for FOC controllers (FTC outputs); (**c**) reference, real, measured speeds; (**d**) reference rotor slip; (**e**) sensor fault indications.

The second experiment considers a disconnection of current sensors that occurs at 2 s in phase A, as shown in Figure 13a. The feedback current signal in phase A is lost, and the TDO-based current diagnosis algorithm recognizes an abnormal state of the current sensor in phase A immediately, and then the current diagnosis algorithm based on the reference RSL is activated to confirm a certain fault of the current sensors. Figure 13d presents the reference RSL, which contains periodical oscillations after losing the phase A current signal in the same way as in the relevant simulation results. Finally, the current diagnosis algorithm correctly detects the failure type and location of the faulty current sensor by simultaneously issuing two flags, the failure location phase A, and the current sensor fault, as shown in Figure 13e. The execution time of the diagnosis algorithm takes only 2.0 ms, corresponding to 20 processing cycles of TMS320F28335, which is a 100 µs/cycle.

**Figure 14:**
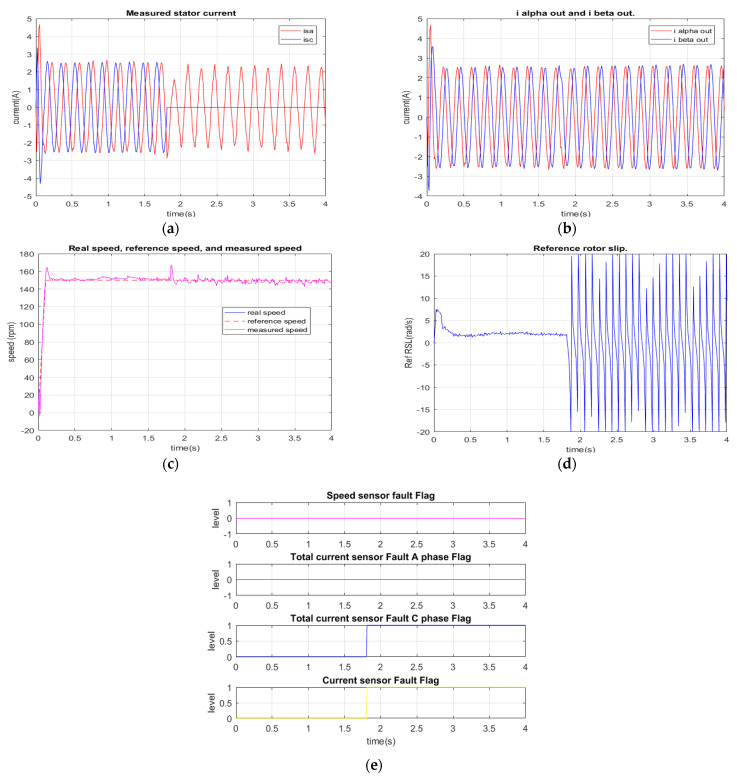
Experimental results: total C phase current sensor failure at 1.8 s. (**a**) Measured currents; (**b**) stator current vector components for FOC controllers (FTC outputs); (**c**) reference, real, measured speeds; (**d**) reference rotor slip; (**e**) sensor fault indications.

Figure 14 describes the response of the current diagnosis algorithm for the current sensor failure at phase C. In this experiment, the lack of the phase C current signal activates the current diagnosis algorithm at the time of 1.8 s. The exact failure type and location of the faulty sensor are quickly determined after 2 ms only. Similarly to the previous experiment, the actual signal of the reference RSL is periodically oscillated by the effect of losing the phase C current signal, as shown in Figure 14d. Finally, two flags, the failure location at phase C, and the current sensor fault are simultaneously issued by the FTC unit, as shown in Figure 14e.

This part describes the performance of the proposed fault diagnosis algorithm for the real induction motor drive, where the experiments were set up in the same scenarios as the relevant simulations in the previous section. Both simulation and experimental results clearly demonstrate the capability and the superior of the proposed approach for this specific application. Moreover, the proposed diagnosis strategy is effective against various insufficient cases of the measuring system of IMDs in low-speed regions.

## 6. Conclusions

The proposed sensor fault diagnosis method has demonstrated its effectiveness in dealing with both speed and current sensor failures. A new idea of using the rotor slip combined with the TDO has been proposed to precisely detect and locate the current sensors faults during the induction motor drive operation. In order to enhance reliability and avoid confusion due to the influence of random noise, an appropriate diagnosing strategy has been recommended where the sequentially specific order determines each fault type in the fault diagnosis unit. The disconnection of the current sensor, which is one of the most severe faults, can be quickly determined by the current sensor diagnosis function within 2 ms. Additionally, the speed sensor diagnosis function can reliably detect the inaccuracy of the speed sensor signal without being interfered with by random noises within 3–3.5 ms. The proposed diagnosis algorithm is more effective and convenient than other existing diagnosis methods. As a result, the IMD’s control system can operate more efficiently due to the lower calculation burden in the same operating conditions. Both simulation and practical experiments have proved the capability of the proposed method.

## Figures and Tables

**Table 1:** Diagnosis function.

Indication Flags	Diagnosis State
FA_Flag = 0, FB_Flag = 0, FW_Flag = 0	Healthy
FA_Flag = 0, FB_Flag = 0, FW_Flag = 1	Speed sensor fault
FA_Flag = 1, FB_Flag = 0, FW_Flag = 0	Total A phase current sensor fault
FA_Flag = 0, FB_Flag = 1, FW_Flag = 0	Total B phase current sensor fault

**Table 2:** Induction motor parameters.

Symbol	Quantity	Value (Unit)
P	Rated Power	2.2 (kW)
U	Rated Voltage	400 (V)
ωr	Rated speed	1420 (rpm)
In	Rated current	4.85 (A)
Rs	Stator resistance	3.179 (Ω)
Rr	Rotor resistance	2.118 (Ω)
Lm	Mutual inductance	0.192 (H)
J	Moment of inertia	0.047 (Kg m^2^)
p	Pole pair number	2
Ls	Stator inductance	0.209 (H)
Lr	Rotor inductance	0.209 (H)

## Data Availability

The data used to support the findings of this study are available from the corresponding author upon request.

## Abbreviation

Cur_sl	Current sensorless
DSC	Digital Signal Controller
FTC	Fault-Tolerant Control
PFTC	Passive Fault-Tolerant Control
AFTC	Active Fault-Tolerant Control
FOC	Field-Oriented Control
IM	Induction motor
IMD	Induction motor drive
Sp_sl	Speed sensorless

## Nomenclature

$i_{S}^{S}$	Stator current vector in [α β] coordinate
$i_{R}^{S}$	Rotor current vector in [α β] coordinate
$u_{S}^{S}$	Stator voltage vector in [α β] coordinate
$u_{a},u_{b},u_{c}$	Stator voltage components in [a b c]
